# Effect of Green Plants on Individuals’ Mental Stress during the COVID-19 Pandemic: A Preliminary Study

**DOI:** 10.3390/ijerph192013541

**Published:** 2022-10-19

**Authors:** Tao Liu, Lin He, Wenhuan Yu, Thomas Freudenreich, Xianhao Lin

**Affiliations:** 1 School of Health, Fujian Medical University, Fuzhou 350122, China; 2 School of Management, Shanghai University, Shanghai 200444, China; 3 School of Management, Zhejiang University, Hangzhou 310058, China; 4 Institute for International Marketing Management, Vienna University of Economics and Business, 1020 Vienna, Austria

**Keywords:** COVID-19 pandemic, green plants, growth status, mental stress, positive emotion

## Abstract

The COVID-19 pandemic has not only jeopardized people’s physical health, but also put additional strain on their mental health. This study explored the role of indoor natural elements (i.e., green plants) in relieving individuals’ mental stress during a prolonged stressful period. A pilot and three formal studies examined the effect of indoor green plants placed in living and working environments on people’s perceived stress during the pandemic and further uncovered its underlying mechanism emphasizing a mediating role of emotion. The pilot study confirmed that the severity of the pandemic positively correlated with individuals’ level of stress. Study 1 then demonstrated that indoor green plants in people’s living environments might reduce their perceived stress during the pandemic, which is referred to as the “plant effect”. Study 2 repeated the plant effect in a field experiment conducted in a working environment and Study 3 revealed a mediating role of positive emotion. This study provides preliminary evidence for the mitigating effect of indoor green plants on individuals’ mental stress during the COVID-19 pandemic period. The indoor green plants placed in living and working environments may elicit positive emotion, which in turn reduce people’s mental stress. In addition, our results reveal that growth status of the indoor green plants affected the plant effect as well.

## 1. Introduction

Mental health refers to states of affirmative health, emotional resilience, and psychological well-being [1], and thus is critical for people’s happiness and social harmony. However, there are plenty of stressful events in life that can harm people’s mental health [2]. In order to relieve stress, many people get close to nature in their spare time and gain spiritual comfort from nature [3,4]. Past studies have repeatedly shown that both indoor and outdoor natural environments have benefits for mental health, such as promoting positive emotion [3] and relieving stress [4]. For instance, in a medical environment, close contact with elements of natural environments may effectively reduce negative emotions such as anxiety and depression in patients [5]. In this line of research, however, researchers have mainly induced mental stress through different experimental manipulations in order to examine the stress-coping effect of natural elements [2,6]. This study aimed to examine the effect of indoor natural elements (e.g., green plants placed in living or working environments) on people’s mental stress during a real, prolonged stressful period and further to discuss the role of growth status of the indoor green plants.

The COVID-19 pandemic provides a natural scenario for research on the effect of a prolonged stress period and relevant coping mechanism. The outbreak of the COVID-19 pandemic, lasting over 2 years, has affected the lives of billions worldwide. In addition to causing economic upheaval and political unrest, the pandemic poses a major challenge to public health. This involves not only physical harm from the disease itself, but also mental issues including increased levels of stress [7]. In order to contain the viral spread, governments across the world have enacted strict measures, placing millions of people in isolation [8,9], which exerted additional strain on people’s psyche [2,10]. Following the line of research focusing on the restorative effect of exposure to nature on people’s wellbeing, one potential solution of relieving stress involves the treatment of placing indoor green plants in people’s living or working spaces. This research may underline the connection between indoor natural environments and human psychological well-being, and provide preliminary evidence for the effect of indoor green plants, the plants having good growth status in particular, on restoring people’s mental health in prolonged stressful periods. 

## 2. Literature Review

Nature has long been seen as a place to purify the mind and relieve stress. Ample research demonstrates that natural scenes and ecological environments effectively reduce individual psychological stress and enhance positive emotions [11,12,13,14,15]. For instance, in Kaplan’s Attention Restoration Theory, nature is central to the argument for restorative experiences, and further points out that restorative environments should have four characteristics: fascination, a sense of being away, extent and compatibility [13]. In this vein, Hartig et al. (2003) showed that walking in a nature reserve can evoke positive emotions and reduce anger; while walking in an urban environment can cause opposite states [16]. Ulrich and his colleagues (1983) also found that exposure to a natural environment improves individuals’ psychological state compared to an urban environment. The physiological measures (i.e., heartbeat cycle, pulse, skin conduction, and muscle tension) further revealed that the natural environment can mobilize the parasympathetic nervous system, relieve stress, and restore calmness [3].

Nevertheless, enjoying nature does not necessarily mean being fully immersed in the natural environment. Sometimes just being exposed to natural elements can benefit an individual’s mental health. Ulrich (1985) proposed the Stress Recovery Theory and highlighted three elements in regard to natural environments that can stimulate positive emotion and relieve stress. These include non-threatening landscapes, green plant elements, and natural-specific elements [3,17]. More recently, Ambrose et al. (2020) found a positive association between home gardening and emotional health when measuring people’s emotional well-being as they engaged in daily activities [18]. Similarly, Zhang et al.’s (2020) study also showed that plants and lighting may have a positive impact on individuals’ emotional state during prolonged isolation [19]. In sum, previous studies suggest that exposure to green plants may improve people’s emotional well-being, whether indoors or outdoors [11,20,21]. 

Based on this line of research, more and more researchers have begun to focus on the stress-coping function and therapeutic value of plants in various indoor or outdoor settings (e.g., gardens, schools, offices, and hospitals) for different groups, including children [22], adolescents [23,24], adults [25,26,27,28], elderly [29], and hospitalized patients [30]. For example, Ali Khan et al. (2016) investigated the impact of green plants on patients and found that patients in wards with green plants were significantly more optimistic and had higher expectations for subsequent health improvements than patients in wards without green plants [31]. This suggests that the presence of plants can make patients have better expectations for postoperative recovery and more positive emotions during hospitalization. This method of achieving therapeutic effects through exposure to green plants and activities related to green plants (i.e., planting, pruning, etc.) is called “horticultural therapy” [32].

Lohr, Pearson-Mims, and Goodwin (1996) also confirmed that green plants may have stress-reducing properties in windowless workplaces. They found that in a windowless indoor space, people with plants displayed greater stress relief (i.e., lower blood pressure levels) and higher levels of productivity (i.e., faster reaction times) in computer tasks than those without plants [33]. Many studies have gone further and suggested that simple visual or other types of sensory presentation may have a positive impact on people [34]. Likewise, cognitive neuroscience research has demonstrated that both pictures of natural scenery and the smell of fresh roses are associated with positive emotions and increased levels of activation in specific brain areas involved in emotional processing [35,36].

Therefore, compared with controls without natural elements or urban environments, previous studies have demonstrated that whether it is being in nature, viewing nature-related photos, or adding natural elements (i.e., indoor green plants) to living or working environments, can be effective measures to deal with problems of mental stress. However, these studies have predominantly examined the temporary effect of plants on people’s mental health; whether the plant effect is effective or not during a prolonged stressful period is still to be determined. Besides, it is also worth noting that the research that focuses on the effect of green plants did not consider the physical characteristics of plants (especially in indoor settings), such as plants’ growth status.

The present research thus aimed to answer these questions with a pilot study and three formal studies. Based on the findings of green plants and its coping effect with mental stress, we hypothesized that placing indoor green plants in living and working spaces would enhance people’s positive emotion, thereby reduce psychological stress in a prolonged stressful period caused by the COVID-19 pandemic. Furthermore, according to Kaplan’s Attention Restoration Theory, fascination is one of the critical characteristics of restorative experience [13]. Plants which grow better are normally seen as more attractive; we thus hypothesized that, during the pandemic, growth status of indoor green plants would positively affect people’s level of mental stress.

The purpose of the pilot study was to verify an important premise of the present research, that is, the more serious the COVID-19 pandemic is, the higher psychological stress people have. Only in this case, the pandemic could be used to provide a prolonged, natural stressful scenario. Then, Study 1 examined the relationship between the presence of indoor green plants (including their growth status) and people’s stress level during the COVID-19 pandemic. Study 2 aimed to repeat the findings of Study 1 in a real-life scene and improve the ecological validity of the study. Study 3 further explored the underlying mechanism of the green plant effect, emphasizing a critical role of positive emotion.

## 3. Materials and Methods

The present research took indoor green plants in people’s living and working environments during the COVID-19 pandemic as research objects and conducted a pilot and three formal studies to explore the impact of indoor green plants on individuals’ mental stress and its underlying mechanism. The participants who did not pass the attention check while finishing questionnaires were excluded in all the studies. To improve validity of the findings, we conducted three studies in samples with different sociodemographic characteristics. The pilot study confirmed that the severity of the COVID-19 pandemic did impact individuals’ mental stress. Study 1 then took Chinese college students as research samples and explored the stress-coping effect of indoor green plants placed in their living environments during the pandemic. Study 2 was a field study, in which arrangement of indoor green plants was manipulated in two office spaces of a company in July 2020. Study 2 repeated the promoting effect of indoor green plants on individuals’ mental stress in a real working environment. Study 3 further revealed the mediating role of positive emotion. The respondents in all studies gave their consent before filling in questionnaires, and all studies were approved by the Institutional Review Board of Zhejiang University.

### 3.1. Pilot Study

#### 3.1.1. Measures

To examine whether the COVID-19 pandemic influences individuals’ mental stress, we conducted a pilot study through an online questionnaire.

In May 2020, we recruited a total of 359 members of the Chinese general population (155 females; average age = 28.25 ± 6.67; average monthly income = 9421.27 ± 10,134.82 CNY) via Credamo (a Chinese online survey platform, which allows account registration only when registrants are more than 18 years of age). The respondents who did not pass the attention-check questions were excluded automatically by the Credamo system. The respondents came from 26 provinces and municipalities (93 cities) across the country. During the data collection period, there were varying numbers of confirmed cases of COVID-19 in these cities and regions.

All respondents were invited to complete an online questionnaire to assess their stress level during the COVID-19 pandemic, especially between February and May 2020. The questionnaire used an updated version of the Perceived Stress Scale with 10 items (α = 0.725), such as “in the past three months, how often have you been troubled by unexpected events?” (1, never; 5, very often) [37,38]. After that, we collected demographic information of the respondents, such as gender, age, monthly income, and education level.

To measure severity of the COVID-19 pandemic in different cities, the cumulative number of confirmed cases in each province and city during May 2020 was collected from the official website of the National Health Commission of the People’s Republic of China. 

#### 3.1.2. Results

Firstly, focusing on the cumulative number of confirmed COVID-19 patients in each city and province (M cities ± SD cities = 786.73 ± 5278.24 cases; M provinces ± SD provinces = 2869.77 ± 11071.90 cases), we calculated the normalized mean value of the number of confirmed cases in each province and city, where each respondent was living in during the pandemic. It was then used as an indicator of the COVID-19 severity (M #xB1; SD = 0.03 ± 0.12) in each respondent’s living environment.

We then examined the relation between COVID-19 severity and individuals’ perceived stress. As Table 1 shows, the result of Pearson correlation analysis revealed a positive correlation between the severity of the COVID-19 pandemic and perceived stress (r = 0.138, *p* = 0.009). Since income was the only category that showed significant correlation with respondents’ perceived stress, income was thus taken as a covariate in subsequent regression analysis. Consistently, the linear regression results still showed that the COVID-19 severity significantly predicted respondents’ perceived stress (Adjusted R2 = 0.048, β = 0.599, *p* = 0.014). The pilot study confirmed that the COVID-19 severity did impact individuals’ perceived stress. 

### 3.2. Study 1

#### 3.2.1. Measures

The COVID-19 pandemic significantly disturbs college students’ daily study and life, and the prolonged duration of isolation and online-study may exert heavy mental stress. To examine the stress-coping effect of indoor green plants, we first recruited 217 student respondents (including 137 females; average age, 22.89 ± 3.13 years) via the school Bulletin Board System (BBS) of Zhejiang University at the end of May 2020 and invited them to complete an online questionnaire. The questionnaire consisted of three parts:

First, all respondents were asked to report whether there were indoor green plants in their living environments during the pandemic (1 = yes; 0 = no). Those who reported ‘yes’ were further asked to evaluate growth status of the indoor green plants on a 5-point scale (1 = very poor; 5 = very good). In the second part, we measured respondents’ level of stress during the pandemic using the same Perceived Stress Scale as used in the pilot study (α = 0.733) [38]. Finally, the respondents reported their demographic information, including gender and age.

#### 3.2.2. Results

We firstly examined the plant effect on respondents’ perceived stress during the pandemic. According to their self-reported result on the item of “whether there were indoor green plants in your living environment during the pandemic”, the respondents were divided into two groups, a group with indoor green plants (*n* = 130) and a group without indoor green plants (*n* = 87). As shown in Table 2, the Spearman correlation analysis revealed that the respondents’ perceived stress during the pandemic negatively correlated with the plant group (rho = −0.155, *p* = 0.022) but positively correlated with gender (rho = 0.200, *p* = 0.003). The independent t-test analysis consistently showed lower stress level in the with-plants group (M = 2.75, SD = 0.6) than in the without-plants group (M = 2.91, SD = 0.05; t (215) = −2.12, *p* = 0.035; Cohen’s *d* = 0.291).

Gender was then considered as a covariate in regression analysis, since only gender showed significant correlation with respondents’ perceived stress. The linear regression analysis verified the relationship between the plant group and perceived stress. As expected, after controlling for gender of the respondents, the presence or absence of indoor green plants significantly predicted the respondents’ perceived stress (see Table 3 for details). 

In addition, we also examined the issue of how perceived growth status of the indoor green plants influences respondents’ perceived stress. The Spearman correlation analysis found that there was a negative correlation between perceived growth status of the indoor green plants and the respondents’ perceived stress (rho = −0.247, *p* = 0.005). Taking gender as a covariate, plants’ growth status as the independent variable, and perceived stress as the dependent variable, a linear regression analysis was then conducted. As shown in Table 4, the growth status of the indoor green plants still negatively predicted the respondents’ perceived stress (beta = −0.138, *p* = 0.023). That is, the better the plants’ growth status in the living environment was, the less stress individuals had.

In summary, Study 1 preliminarily demonstrated the coping effect of indoor green plants on individuals’ mental stress. Placing indoor green plants in living environments may reduce individuals’ stress level during the pandemic. In addition, growth status of the indoor green plants may impact validity of the plant effect.

### 3.3. Study 2

#### 3.3.1. Measures

**Figure 1:**
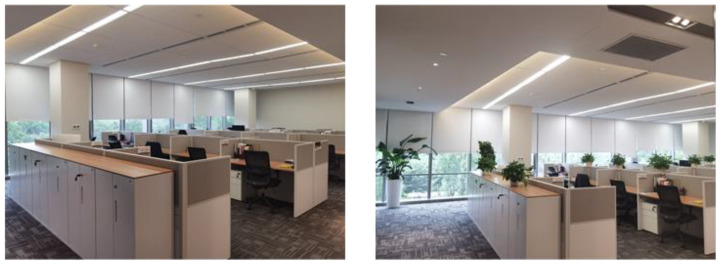
Example of plants placement in field experiment (Study 2).

In Study 2, we conducted a field experiment in a real estate company located in Shanghai, which had just moved to a new office building in July 2020 from Fuzhou city. We placed indoor green plants in one working space but did not in another to manipulate the independent variable of the indoor green plants (see Figure 1). The main purpose of Study 2 was to replicate the main effects in a real-life scenario with different samples to improve the ecological validity of the plant effect.

We selected two working spaces dispersed on different floors (each working space has about 200–300 square meters) to conduct the experiment. Two project teams (‘A’ and ‘B’) working in these spaces were responsible for similar real estate projects during the moment and thus may suffer an equivalent level of work-related stress. 

Team ‘A’ had no indoor green plants in their working space (without-plants group), whereas team ‘B’ had indoor green plants that are normally used in office spaces [33] at the junction of every two adjacent workstations (with-plants group) as shown in Figure 1. Study 2 involved a total of 33 employees between the two teams. There were 16 employees in team ‘A’ (5 females; 18–50 years old; only one employee was over 40 years old) and 17 employees in team ‘B’ (7 females; 18–40 years old). Employees in the two teams finished the questionnaire after working in environments with or without indoor green plants for 20 days (July 10, 2020, to July 30, 2020). The questionnaire used the same Perceived Stress Scale to measure the perceived stress level of the employees (α = 0.810) [38].

#### 3.3.2. Results

The Mann-Whitney U test showed that there was a significant difference in employees’ perceived stress after working in the environment with (or without) plants for 20 days (*p* < 0.001). Consistently, the Spearman correlation analysis also showed a significant correlation between the plant group and the perceived stress (rho = 0.630, *p* < 0.001). People who worked with indoor green plants had a significantly lower level of stress (M = 2.28, SD = 0.66) than those who worked without plants (M = 3.00, SD = 0.23), confirming the plant effect in a real working space.

### 3.4. Study 3

The purpose of Study 3 was to repeat the main effect and further examine the mediating role of positive emotion in a more general population.

#### 3.4.1. Measures

We recruited 417 members of the Chinese general population (186 females; average age 28.64 ± 6.85 years old, average monthly income 9570.97 ± 9254.53 RMB) via Credamo in July 2020. The questionnaire used the same scales as in Study 1 (Perceived stress: α = 0.733). In addition, we added the Positive Affect and Negative Affect Scale (PANAS; Watson et al., 1988) to measure respondents’ emotional status (α = 0.823) [39], and asked the respondents to report to what extent they feel certain emotions during the pandemic period. The scale included 20 specific items, such as distressed, scared, inspired, active, etc. (1 = very slightly or not at all; 5 = extremely).

#### 3.4.2. Results

According to the result of the presence or absence of the green plants in the living environment (1 = yes; 0 = no), we divided the respondents into two groups: with-plants group (*n* = 313, perceived stress: M = 2.50, SD = 0.54; positive emotion: M = 3.27, SD = 0.66) and without-plants group (*n* = 104; perceived stress: M = 2.64, SD = 0.48; positive emotion: M = 2.79, SD = 1.02). The Spearman correlation analysis revealed that the perceived stress negatively correlated with the plant group (rho = −0.137, *p* = 0.005). 

In addition, the perceived stress showed significant negative correlations with respondents’ monthly income (rho = −0.131, *p* = 0.008) and education level (rho = −0.92, *p* = 0.059), but had no relations with their gender (rho = 0.038, *p* = 0.445) and age (rho = −0.057, *p* = 0.246). Therefore, we took monthly income and education level as the covariates, the plant group as the independent variable, perceived stress as the dependent variable, and positive emotion as the mediating variable to examine the mediating effect. Using Model 4 of SPSS Process v3.5, a bootstrap analysis, with samples of 5000 and a 95% confidence interval, was conducted.

**Figure 2:**
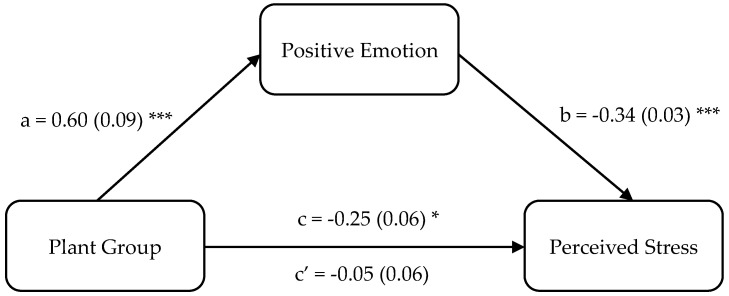
Mediation regression analysis results of the plant group predicting the perceived stress. *, *** represent *p* < 0.05, *p* < 0.001, respectively.

As shown in Figure 2, the direct effect between the plant group and perceived stress was not significant. However, the indirect effect via positive emotion was significant, which indicates that positive emotion fully mediated the plant effect. Having indoor green plants in one’s living environment may promote individuals’ positive emotion, which in turn reduce their perceived stress. Consistent with the results of Study 1, growth status of the indoor green plants negatively correlated with perceived stress (rho = −0.319, *p* < 0.001), but positively correlated with the positive emotion (rho = 0.297, *p* < 0.001). 

## 4. Discussion and Conclusions

Through a pilot and three formal studies, the present research explored the coping effect of indoor green plants on individuals’ mental stress during the COVID-19 pandemic. Two main results may be obtained. Firstly, the research supports the plant effect and reveals that placing indoor green plants in living or working environments may help to reduce people’s psychological stress even in a prolonged pandemic period. In addition, we provide preliminary evidence for the mediating role of positive emotion. Secondly, our findings suggest that growth status of indoor green plants may affect the validity of the plant effect. The plants, especially those that grow well, may promote positive emotion, thereby reducing people’s mental stress during prolonged stressful periods. 

In terms of the theoretical contribution, our research integrates elements of the indoor natural environment, emotional states, as well as psychological stress, revealing an interactive relationship between humans and nature during the COVID-19 pandemic, which constitutes a prolonged stressful environment. Previous literature has consistently revealed the coping effect of natural elements on human psychological well-being in well-designed experiments (e.g., [2,3,4,5,6,11,12,13,14,15]). However, the validity of the plant effect in prolonged stressful environments is not well examined. The COVID-19 pandemic provides a real, prolonged stressful environment; we then confirmed that, even in prolonged stressful environments, indoor green plants could promote individuals’ positive emotion and in turn reduce their mental stress to some extent. People cannot live apart from nature, and a life force indwells in nature. As the Bulgarian Proverb says “Nature, Time and Patience are the three great physicians”. Getting close to nature is of benefit to emotional as well as psychological well-being [13,16].

In addition, the present research calls for attention to subsequent in-depth study regarding the impact of indoor vegetation diversity on human physical and mental health. More important, we shed light on the physical characteristics of indoor green plants in this research, such as growth status, when examining the stress-coping effect of indoor green plants. Previous studies have paid little attention to role of the physical characteristics of natural elements. However, the Kaplan’s Attention Restoration Theory proposes that fascination is a critical characteristic of restorative environments [13]. If a healthy natural environment attracts human beings and serves as a restorative resource [16], then sickly, dried-out plants may prime decay and death and in turn induce negative emotion and increased stress. The present research reveals a significant negative correlation between growth-status of indoor green plants and individuals’ stress level, suggesting that physical characteristics of plants may affect the validity of the coping effect of green plants. 

This research has practical contributions as well, especially considering the ongoing global impact of the pandemic. The results of this research provide people with a new coping strategy in addition to social support. Placing well growing indoor green plants in a living environment and workplace may be a simple and convenient way to cope with mental stress. At the frontline of pandemic prevention, as well as control and isolation areas, indoor green plants could be arranged to relieve people’s mental stress. In addition, through more research on the “psychological effects” of different types of plants, we can consciously place different green plants in various places, such as entrepreneurial parks, hotels, schools, etc., to promote positive interaction between human beings and nature. It is also noteworthy that we should pay more attention to physical characteristics of green plants. It is a common phenomenon that some companies may place indoor green plants in working spaces and offices but have no constant care. In this case, the sickly, dried-out plants may reversely impair individuals’ psychological well-being.

This research also has certain limitations. The current research aimed to explore the impact of green plants on relieving mental stress during the COVID-19 period. There were several aspects concerning variable measurements and sample size that needed to be further discussed in future studies, in order to reach more concrete conclusions about the plant effect. First, we measured the plants’ growth status in the living environment by respondents’ subjective evaluations, instead of manipulating or objectively measuring the growth status of green plants. In addition, we did not manipulate the growth status of green plants in one experiment to confirm the validity of the effect. In future research, the growth status of the indoor green plants should be manipulated and it can be further cross-integrated with the field of botany to objectively measure the growth status of green plants. Even the type and intensity of pheromones released by different types of green plants, as well as the visual and tactile stimuli they release, can be considered when examining the interaction between plants and humans. Second, some screening measures, such as social support, the levels of anxiety and depression, might not be fully considered in this research. Even though, in Study 1 and Study 2, the samples were college students at Zhejiang University and company employees, none had any diagnoses of mood disorders or other disorders (including COVID-19 disease). In future research, more factors will be taken into consideration to avoid the confounding effect of mood in the perception of stress during the pandemic. Third, despite that this research involved nearly 700 respondents in total and included a field experiment, the sample size was still insufficient to identify individual and environmental differences. In future research, more refined analysis and research can be carried out for specific populations in more specific settings. Fourth, this research did not consider the impact of individual characteristics such as the levels of anxiety and depression, which may also influence the perception of stress. More studies are needed to examine the interactions between indoor green plants and human psychological well-being, considering both characteristics of plants and individuals. Fifth, in Pilot Study, Study 1 and 3, the respondents reported their perceived stress level or affect during the pandemic based on their memories when the pandemic was quite severe, which may lead to certain subjective biases. Future studies may obtain participants’ objective performance or take advantage of physiological measurements, such as heart rate and skin conductance, to measure affect and stress levels more objectively [40]. Fifth, during the time the field experiment was conducted, although there were still sporadic new cases, the pandemic had generally been contained in China. Future studies are needed to investigate whether indoor green plants have a positive impact on relieving stress in order to provide more practical evidence for promoting mental health among people suffering from prolonged stress. Sixth, in Study 2 both ‘team A’ and ‘team B’ were not very numerous and their individual characteristics were also unknown (e.g., environment preferences etc.). To avoid biases future study could perform the experiment as follows: ‘team A’ works for 20 days in the working space with indoor plants while ‘team B’ without plants and then they switch places. Finally, due to the sudden outbreak of the COVID-19 pandemic, these studies all have a limitation from a lack of the baseline of people’s mental stress before the outbreak of the pandemic.

In sum, this research was designed to provide preliminary evidence of the plant effect on mental stress during prolonged stressful periods. Also, we hope that the current research will draw attention to exploring means of maintaining mental health during stressful events such as the COVID-19 pandemic and to focusing on more detailed characteristics of indoor green plants while considering green plants as a stress-coping means.

## Figures and Tables

**Table 1:** Correlation matrix of variables.

Variables	1	2	3	4	5	6
Gender	1.00					
Age	0.167 **	1.00				
Income	−0.06	0.312 **	1.00			
Education	0.08	0.212 **	0.397 **	1.00		
Stress level	0.09	−0.03	−0.177 **	−0.07	1.00	
COVID-19 severity	−0.05	−0.08	−0.06	0.03	0.138 **	1.00

Note: ** represent *p* < 0.01.

**Table 2:** Correlation matrix of variables.

Variables	1	2	3	4	5
Gender	1.00				
Age	−0.13	1.00			
Group	0.12	0.09	1.00		
Stress	0.20 **	−0.02	−0.16 *	1.00	
Growth status	0.12	0.05	0.90 **	−0.22 **	1.00

Note: *, ** represent *p* < 0.05 and *p* < 0.01, respectively.

**Table 3:** Regression analysis results of the plant group (with-plants vs. without-plants) predicting perceived stress.

Variables	Perceived Stress	
	Model 1	Model 2
	Beta	Beta
Constant	2.437 ***	2.132 ***
Gender	0.201 **	0.221 **
Plant group		−0.192 *
F	9.065 **	7.875 **
R^2^	0.040 **	0.069 *

Note. *, **, *** represent *p* < 0.05, *p* < 0.01, *p* < 0.001, respectively.

**Table 4:** Regression analysis results of plant growth status predicting perceived stress.

Variables	Perceived Stress	
	Model 1	Model 2
	Beta	Beta
Constant	2.349 ***	2.863 ***
Gender	0.200 **	0.206 *
Plant growth status		−0.138 *
F	5.314 **	5.407 ***
R^2^	0.040 **	0.078 **

Note. *, **, *** represent *p* < 0.05, *p* < 0.01, *p* < 0.001, respectively.

## Data Availability

The data presented in this study are available on request from the corresponding author. The data are not publicly available due to requirement of approval of the Institutional Review Board.
